# Development of a Chemiluminescence Assay for Total N-Terminal Propeptide of Type I Collagen and Its Evaluation in Lung Transplantation

**DOI:** 10.1155/2022/2711414

**Published:** 2022-01-10

**Authors:** Shuang Han, Fang Gong, Yifeng Xue, Chunxin Wang, Xiaowei Qi

**Affiliations:** ^1^The Affiliated Hospital of Jiangnan University, Department of Pathology, Wuxi, China; ^2^School of Medicine, Jiangnan University, Wuxi, China; ^3^The Affiliated Wuxi People's Hospital of Nanjing Medical University, Department of Laboratory Medicine, Wuxi, China

## Abstract

Serum P1NP, one of the important biomarkers for bone turnover, is commonly used for the prediction of bone fracture and the prognosis of osteoporosis after therapy. We developed a P1NP chemiluminescence assay and evaluated changes in bone metabolism markers in lung transplant patients. The screened 2 P1NP antibodies with constructed antigens and *α*-1 chain antigens expressed by the *Corynebacterium glutamate* expression system were applied into assay development. The assay performance was evaluated to examine the reliability. A normal Q-Q plot was used to establish male reference interval. Changes of bone metabolism markers before and after lung transplantation in 19 patients were evaluated. The linear factor *R* of P1NP reagent was greater than 0.99. The limit of detection was 3.32 ng/ml. The precision of the three batches of P1NP reagents was lower than 8%. Method comparison with Roche P1NP reagent showed that the correlation coefficient *R*^2^ was 0.91. In the monitoring of bone mass in a short time, bone metabolism markers can better indicate the change of bone mass, while the traditional bone mineral density detection is lagging behind the bone metabolism markers. P1NP and *β*-CrossLap to bone mass change in patients after lung transplantation, and P1NP and *β*-CrossLap are very good clinical markers for bone mass monitoring.

## 1. Introduction

Serum P1NP, introduced by the IOF-IFCC Bone Marker Standards Working Group, is one of the key biomarkers indicating bone formation. Indeed, human serum or plasma P1NP level was indicative for either the prediction of bone fracture or the monitoring of prognosis of osteoporosis patients after receiving therapies. Osteoporosis is one of the most prevalent metabolic bone diseases and is especially prevalent among elderly people worldwide. Low bone mass, measured as bone mineral density (BMD), is often associated with increased risk of bone fracture and is the leading cause of morbidity and mortality of osteoporosis [1]. In addition, bone turnover markers (BTMs) are parameters that were also widely measured in metabolic bone diseases, including osteoporosis, to evaluate the severity of the disease. Because of the significance in studying the bone health, numerous assays were developed and are currently available [2].

Type I collagen is an important component of the bone matrix, and osteoblasts secrete its precursor procollagen molecule during bone formation [3]. In 2012, the IFCC-IOF Working Group recommended that P1NP and *β*-CrossLap (also called *β*-CTX) should be used as reference markers for evaluating bone turnover and thus started the standardizations of the clinical assays that could provide solid results of the serum/plasma *β*-CrossLaps and P1NP during clinical practice [3]. Since then, marked progress have been made in studying P1NP and other biomarkers, including vitamin D, BAP, PICP, and *β*-CrossLaps in the field of bone health [4–6]. These studies also suggested that BTMs are essential markers and are independent of BMD, and the results of both are complimentary.

P1NP is composed of a trimeric structure derived from collagen, which has both N-amino (P1NP) and C-carboxy terminal extensions (P1CP) in its chemical structure [7–9]. However, these extensions (propeptides) can be cleaved by enzymes during bone matrix formation and are either released into the circulation or trimmed into a monomeric form by the thermal degradation effect [10]. Generally, the propeptide measured by assays is from the aminoterminal because P1NP procollagen-type 1 N-terminal-propeptide is released during type 1 collagen formation and its subsequent incorporation into the bone matrix and thus may be defined as a true bone formation marker [11]. P1NP is a trimer. Each P1NP molecule consists of two identical *α*1 chains and one *α*2 chain, in which the *α*1 chain consists of 161 amino acids and the *α*2 chain consists of 79 amino acids. The three strands are wound around each other to form a specific trihelical conformation, but in vivo, the P1NP trimer quickly forms a single-stranded *α*1 chain due to thermal degradation, so in vivo, the form of P1NP is mainly trimer and *α*1 chain. However, commercial detection assays detect both fractions present in blood and are therefore called total P1NP. Enzyme linked immunosorbent assay (ELISA) and chemiluminescence immunoassay (CLIA) are two frequently employed tests for quantification of P1NP in clinics. The traditional quantitative method is sandwich ELISA [12–14] with lower cost and easier to be developed and convenient to use. But the immunoassay method has been greatly developed in recent years [15, 16]. For P1NP measurement, chemiluminescence immunoassay seems to be widely used in clinics due to its great advantages in sensitivity and dynamic range [17]. Besides, CLIA is automatable and just need small volume sample in comparison with classic ELISA, thus making it very popular in clinical application [18–21].

Previous studies have shown that the peripheral level of total P1NP was significantly changed upon antiresorptive as well as anabolic therapy [22] within months from the start of treatment. A suboptimal response to treatment indicates noncompliance or the presence of secondary causes of osteoporosis that required further examination. Other studies on women with postmenopausal osteoporosis [23] reported that P1NP is indicative for monitoring the efficacy of treatments and predicting fracture risk. Moreover, creating reference intervals for BTMs is critical for interpreting the results from osteoporosis patients, particularly in combination with clinical treatment for individual patients [3]. The measurement of very high BTMs values during the initial assessment of patients with osteoporosis suggests that comorbidities such as metabolic abnormality or malignancy is involved. Therefore, it is necessary to establish reference intervals for healthy men and women aged 30–45 years. Osteoporosis is one of the common comorbidities among patients who have undergone solid organ transplantation, particularly after the first year [24, 25]; however, few studies have fully evaluated BTMs for these patients, even though BTMs could guide the selection of appropriate treatments.

In this study, we aimed to develop and evaluate the performance of a chemiluminescence assay for total P1NP able to detect intact molecules in both their monomeric and monomeric forms. Subsequently, the clinical evaluation of P1NP, 25-VITD, *β*-CrossLap, and OSTEOC were studied, in particular at the different stages of postlung transplantation. Osteoporosis is a well-recognized complication of lung transplantation that reduces the quality of life after transplantation. P1NP is generally used for monitoring osteoporosis or monitoring the prognosis of patients with bone metabolic diseases. Few studies have been conducted on the changes of P1NP in other diseases, especially osteoporosis as a complication after organ transplantation. In lung transplant patients, osteoporosis is a necessary complication due to the large amount of immunosuppressant used during and after the operation, so patients must take calcium supplementation to slow down the rate of osteoporosis. However, the current postoperative monitoring of the patient's bone mass is still mainly based on bone mineral density detection, which cannot timely reflect the changes in the patient' bone mass. Therefore, our study is the first to evaluate changes in bone metabolic markers in lung transplantation patients. Our preliminary results provide insights into the potential clinical treatment of affected individuals.

## 2. Materials and Methods

### 2.1. Reagents and Materials

Dynabeads MyOne streptavidin-precoated beads, EZLink Sulfo-NHS-LC-Biotinylation Kit, succinimidyl 4-(N-maleimidomethyl) cyclohexane-1-carboxylate, and 4′-hydroxyazobenzene-2-carboxylic acid (HABA) solution were obtained from Thermo Fisher (Waltham, MA, USA); perfluorohexanoate, methanol, EDC, and NHS; horseradish peroxidase (HRP) was purchased from BBI Solutions (Portland, ME, USA); streptavidin was purchased from Hangzhou NeuroPeptide Biological Science and Technology Incorporation, Ltd. (China). The AKTA purifier system was purchased from GE Healthcare (Chicago, IL, USA); microscopes were purchased from Olympus (Tokyo, Japan); and an automagnetic beads chemiluminescent analyser was supplied by Baiming Biotechnology (Yuncheng, China). Antigens were purchased from Sigma-Aldrich (St. Louis, MO, USA).

### 2.2. Source of Raw Materials

We designed three peptide fragments peptide-1: QEEGQVEGQDEDIPITC, peptide-2: CGVEGPKGTGPRGPRG, peptide-3: CGPPGPPGPPGPPGLGGN and immunized mice to obtain P1NP monoclonal antibody. The P1NP-*α*1 chain was expressed by the *Corynebacterium glutamate* expression system. Natural P1NP protein was purified from the pleural effusion of untreated tumor patients.

### 2.3. Chemiluminescence Assay Development

**Scheme 1:**
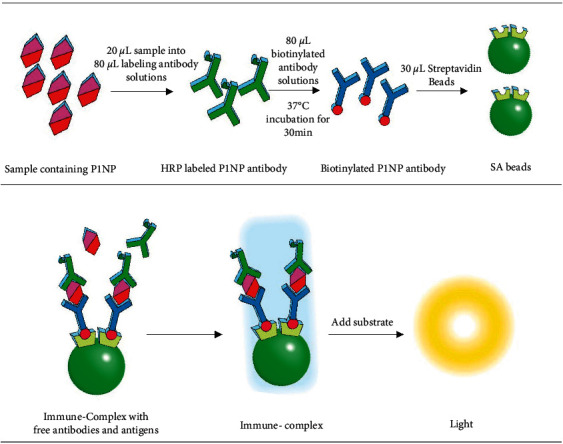
Schematic representation of P1NP chemiluminescence assay. The assay uses one-step sandwich format for P1NP quantitative measurement of P1NP: 20 *μ*L sample was added into 80 *μ*L labeling antibody solutions and followed by adding 80 *μ*L biotinylated antibody solutions; after incubation for 30 min at 37°C, 30 L streptavidin beads were added into complex. The immune complex was then separated and washed 3 times. Substrate was added to read the RLU value. The RLU value represent the concentration of P1NP in sample.

We followed the methods of Shuang Han et al., 2020 [24, 25]. Dynabeads MyOne were washed twice with 25 mM 2-morpholinoethanesulfonic acid (MES) buffer (pH 6.0) to remove the storage buffer from the beads. EDC and NHS solutions were added to the Dynabeads to activate binding on the particle surface, suspended particles, and well-mixed compounds. The mixture was incubated with gentle tilt rotation at room temperature for 30 min. The beads were then washed two more times to remove the supernatant. The necessary amount of streptavidin was added to preactivated magnetic beads and incubated for another 30 min at room temperature with gentle tilt rotation. Last, the beads were washed twice again and suspended in storage solution with PBS buffer containing 0.5% BSA, 0.05% polysorbate 20, and 0.02% sodium azide. The assay schematic illustration for P1NP Chemiluminescence assay is shown in Scheme 1.

The biotin-streptavidin system was used to test the quality of the prepared magnetic beads. Two sets of reaction tubes were prepared and added 30 *μ*L of SA-coated magnetic beads. Then, 50 *μ*L of biotinylated HRP (50 ng/mL) was added to one tube and 50 *μ*L of HRP (50 ng/mL) was added to the other. Both tubes were incubated for 30 min at 37°C, followed by washing the beads 3 times with washing buffer. Finally, 100 *μ*L of substrate reagent was added to generate signals. The RLUs were 710,325 with biotinylated HRP and 352 with HRP.

Different amounts of P1NP antibody and EDC were dissolved in separate 0.05 M sodium bicarbonate solutions, mixing to dissolve completely. HRP was dissolved in 0.1 M phosphate buffer (pH 7.2). The resulting mixed solution was added to the HRP solution, and the pH was lowered to pH 5.8 using diluted hydrochloric acid. Then, the mixture was incubated for 5 h at room temperature. Finally, the impurities were removed using a desalination column.

The P1NP antibody-HRP conjugate was prepared at different antibody/HRP ratios. Biotin-P1NP-SA magnetic particle coupling was used as a probe to test the quality of the anti-P1NP antibody-HRP conjugate. Several sets of reaction tubes were prepared: 30 *μ*L of biotin-P1NP-SA magnetic particle coupling and 50 *μ*L of different anti-P1NP antibody-HRP with HRP as a control. The tubes were incubated for 30 min at 37°C and then washed 5 times using washing buffer. Next, 100 *μ*L of substrate reagent was added. The results indicate that anti-P1NP antibody-HRP conjugates were ready for use.

### 2.4. Assay Performance Testing

#### 2.4.1. Measuring Range

The limit of blank, limit of detection, and limit of quantitation were determined in accordance with the Clinical and Laboratory Standards Institute (CLSI) EP17‐A2 requirements. In this study, the detection limit was focused, in which 20 repeats of the black sample were tested. The mean ± 2 standard deviations (SD) were defined as the detection limit.

### 2.5. Linearity

Multiple sample types and levels were selected in order to determine the maximum possible measurement interval in the assay's insert. The low sample must be at or below the detection limit. The high sample must be above the highest calibrator that could be produced as a result of the manufacturing or value assignment process.

### 2.6. Accuracy

There is no available WHO or USP materials as P1NP standards. As Roche P1NP assay was the most popular in clinics, so 2 serum samples (one with the highest P1NP value and the other with the lowest P1NP value) were examined with Roche P1NP assay. 3 repeated value was obtained for each sample, and the calculated average value was taken as the theoretical value. The 2 serum samples were aliquoted in certain proportion to make another 3 samples. The 5 samples (with theoretical value T1, T2,…, T5) will be tested with 3 lot of self-developed P1NP assays, 3 repeats for each. The average value was calculated as measured value (M1, M2,…, M5). The bias will be the ratio of (Mn−Tn)/Tn.

### 2.7. Expected Values for Male

Sera taken from 294 healthy men aged 35–65 years interned at The Affiliated Wuxi People's Hospital of Nanjing Medical University were measured for total P1NP levels using the assay developed in this study. Normal distribution and frequency analysis were performed, in which the 5^th^ and 95^th^ percentiles were calculated as the reference range.

## 3. Method Comparison

The methods were compared using the Passing–Bablok method.

### 3.1. Serum Collection and Patient Information of Lung Transplantation

19 samples aged 25–75 years were taken into analysis in this study, and the samples was obtained from The Affiliated Wuxi People's Hospital of Nanjing Medical University. Approval has been obtained from ethics committee before study.

### 3.2. Statistical Analysis

Statistical analysis was performed using Microsoft Office 365, PRISM program version 8.4.3 for Mac (GraphPad Prism, Windows), and SPSS version 26.0 (IBM Co.). One-way analysis of variance (ANOVA) with Bonferroni's multiple comparison post-test and unpaired Student's/Mann–Whitney *t*-test were used.

## 4. Results

### 4.1. Total P1NP Chemiluminescence Assay Development and Performance Evaluation

**Figure 1:**
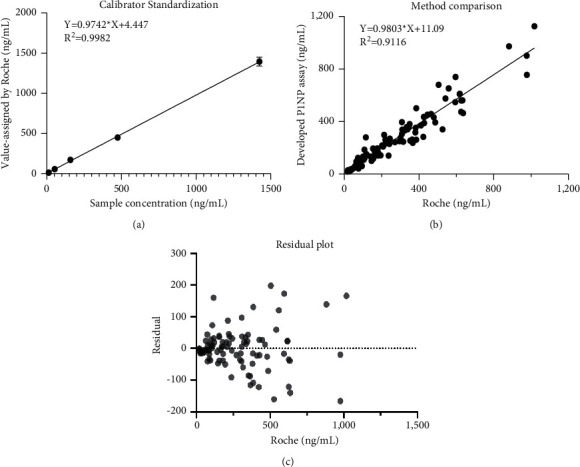
Basic performance evaluation of developed P1NP assay. (a) The calibrator standardization with Roche P1NP assay. The linear correlation of the sample and Roche has a slope of 0.9742. (b) The method comparison of our assay with the Passing–Bablok method between self-developed assay and Roche P1NP assay with slope 0.9803. 94 samples with a range from 15 ng/mL to 1200 ng/mL were included in this study. Dash line means 95% confidence interval. (c) The residual plot analysis of method comparison. The points state the deviation degree of the results obtained from self-developed assay and Roche P1NP assay. The farther the points deviate from zero line means, the larger the deviation exists between the two assays.

The native P1NP proteins obtained from the hydrothorax of cancer patients were used as control samples, while the recombined P1NP proteins were used as calibrators. Three antibodies were captured with beads and conjugated with HRP to obtain a complete kit for P1NP detection. The lower limit and linearity (Supplementary Table 1). (Figure 1(a)) were studied according to the EP guidelines. The calibrators were prepared to a theoretical value by weighing on scales. The value was then verified with the Roche assay. The linearity analysis (Figure 1(a)) shows a good linear correlation with a slope of 0.97. The predicted linearity range was from 5 to 1200 ng/mL, which is consistent with the Roche assay (Figure 1(a)).

Other performance measures, such as precision and accuracy, were also analyzed, as shown in Supplementary Tables 2 and 3.The precision values were obtained from 10 repeated tests of three different concentrations of the P1NP control samples. The CV% number was calculated with a result of not more than 8%. After verifying the assay, method comparison (Figure 1(b)) was evaluated using the Roche Elecsys total P1NP assay, which is the only available assay for commercial use on the market. Passing–Bablok comparison data showed a good correlation between the Roche assay and our own assay, with the correlation coefficient *R*^2^ was 0.91. The residue plot showed that the correlation of the two assays showed a good consistency in the low range (Figure 1(c)).

### 4.2. P1NP-*α*1 Chain Affinity Test

**Figure 2:**
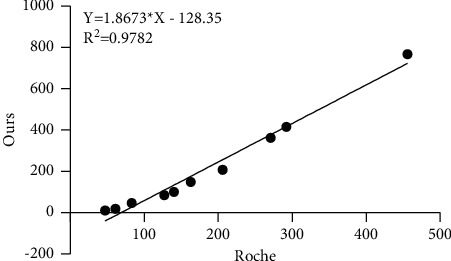
Comparison of test results of P1NP-*α*1 chain.

The P1NP-*α*1 chain expressed by *Corynebacterium glutamate* was diluted to a certain gradient with horse serum and detected simultaneously by the method established in this study and Roche kit, respectively. The linear regression equation with the Roche measure showed that the correlation coefficient *R*^2^ was 0.9781, but the measured value in this study was 1.8 times higher than that in Roche measure, indicating that the affinity of this study method to *α*1 chain was higher than that in Roche measure (Figure 2).

### 4.3. Total P1NP Reference Interval in Males

**Figure 3:**
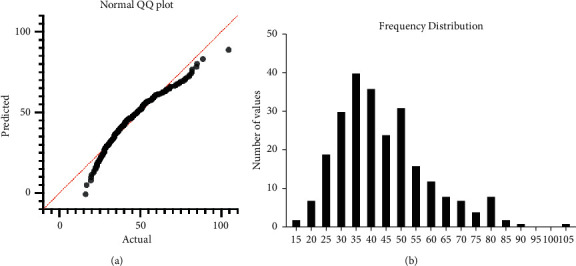
Normal distribution analysis of P1NP results of male. (a) Normal QQ plot analysis. Each point means the deviation from the diagonal (red line). The more the data fall on the diagonal means the more the possibility to meet the normal distribution. (b) Frequency distribution analysis. 248 data were divided into groups; the column volume means the total number of this group.

We collected samples from 248 healthy individual and checked the P1NP value using the assay developed in this study. The values we obtained were analyzed using a normal Q-Q plot analysis (Figure 3(a)) and normal distribution (Figure 3(b)). A Q–Q plot was used to verify whether the data fit the normal distribution. Afterwards, a normal distribution is obtained as shown in Figure 3(b). The 95% confidence interval was taken as the expected value (Table 1), and a reference value of 23.36–77.05 ng/mL was obtained. The 90% CI of lower limit was 21.02 ng/mL and 25.37 ng/mL, while the 90% higher limit was 69.35 ng/mL and 84.76 ng/mL.

### 4.4. The Clinical Connection of Total P1NP Combined with Other Biomarkers in Lung Transplant

**Figure 4:**
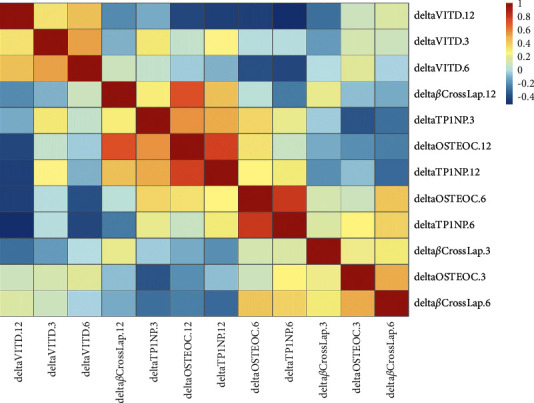
The correlation matrix analysis of 4 bone loss biomarkers. Red means positive relationship and blue means negative relationship. DeltaVITD.3, deltaVITD.6, and deltaVITD.12 mean the delta value tested with VITD assay on point of months 3, 6, and 12. Delta*β*Crosslap.3, delta*β*Crosslap.6, and delta*β*Crosslap.12 mean the delta value tested with *β*-CrossLap assay on point of months 3, 6, and 12. DeltaTP1NP.3, deltaTP1NP.6, and deltaTP1NP.12 mean the delta value tested with P1NP assay on point of months 3, 6, and 12. DeltaOSTEOC.3, deltaOSTEOC.6, and deltaOSTEOC.12 mean the delta value tested with OSTEOC assay on point of months 3, 6, and 12.

In our study, 19 lung transplant patients were observed from pretransplantation, 3 months posttransplantation, 6 months posttransplantation, and 12 months posttransplantation. At each of the four stages, the developed P1NP assay combined with 25-VITD, OSTEOC, and *β*-CrossLap was checked. The relationship of each marker is shown in Figure 4; red denotes a positive relationship, while blue indicates a negative relationship. From the correlation matrix analysis, P1NP showed a positive relationship with OSTEOC and a *β*-CrossLap after 6 months of transplantation, while vitamin D showed a negative relationship with the other three markers.

**Figure 5:**
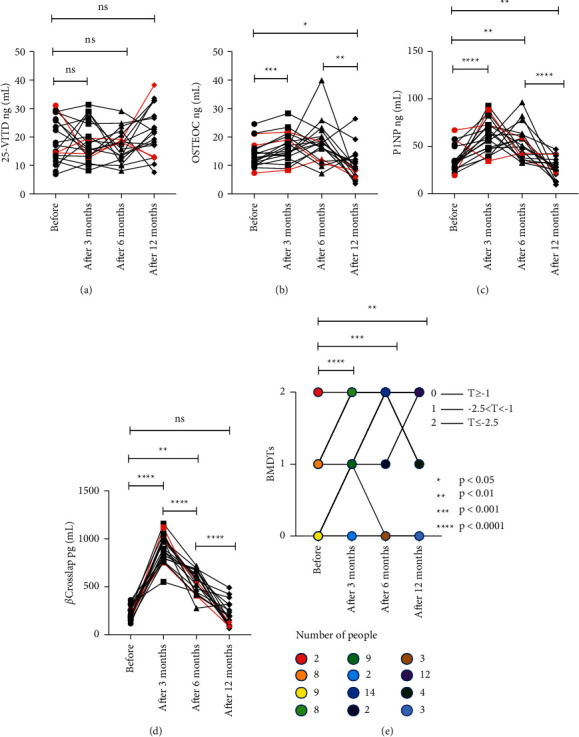
The change of bone loss biomarkers during pre and postlung transplantation at the point of before transplantation, month 3 after transplantation, month 6 after transplantation, and month 12 after transplantation. (a) The change of 25-VITD. (b) The change of OSTEOC. (c) The change of P1NP. (d) The change of beta-CrossLap. (e) The change of BMDTs. 0 means the *T* value ≥−1; 1 means −2.5 <*T* value <−1; 2 means *T* value ≤−2.5. ^*∗*^, *P* value <0.05. ^*∗∗*^, *P* value <0.01. ^*∗∗∗*^, *P* value <0.001. ^*∗∗∗∗*^, *P* value <0.0001. The line in red means female individual.

A further tendency map is shown in Figure 5. Both *β*-CrossLaps and P1NP levels increased during the first 3 months after lung transplantation, while 25-OH VD and OSTEOC levels did not change significantly. After 3 months, both *β*-CrossLaps and P1NP levels decreased significantly, while 25-OH VD and OSTEOC levels remained unchanged. P1NP, OSTEOC, and *β*-CrossLap were all positively correlated 6 months after transplantation, and a decrease in their values was observed after transplantation. However, the changes in BMDTs were not obvious. In conclusion, P1NP, OSTEOC, and *β*-CrossLap are closely associated with BMDTs and bone loss. The underlying mechanisms remain unclear and need to be further investigated, partly due to the use of steroid hormone drugs and a large number of immunosuppressive agents during transplantation. BMDTs were even within the normal range (≥1) in 2 of the 19 lung transplant patients, but the *β*-CrossLap measurements showed very severe bone loss, and the P1NP measurements showed relatively slow bone formation, leading to the formation of osteoporosis, but BMDTs did not suggest osteoporosis. These results suggest that *β*-CrossLap and P1NP may be more sensitive than conventional BMDTs to predict osteoporosis at an earlier stage when osteoporosis occurs. In addition, the correlation matrix showed that 25-OH VD was negatively correlated with the other three markers. 25-OH VD and OSTEOC did not show significant changes before and after lung transplantation, which may indicate that 25-OH VD and OSTEOC are not suitable for monitoring short-term fluctuations in the bone metabolism in vivo. The P1NP and *β*-CrossLap assays may be more suitable for evaluating the treatment of osteoporosis in patients after lung transplantation, providing a continuous monitoring process of bone mass, but more research is needed.

## 5. Conclusion and Discussion

Assays for the N-terminal propeptide of type I procollagen that measure both the trimeric and *α*1 chain in serum are called total P1NP [26]. The first generation of this assay, the manual enzyme-linked immunosorbent assay (ELISA), is outdated technically and not convenient to use due to its complexity, thus less popular in hospitals. The latest assays applied automated electrochemiluminescence immunoassay (ECLIA) on the Elecsys automated immunoanalyzer was first launched in 2004 by Roche Diagnostics. No other assays are currently available in the market as the relevant antibodies and antigens offering high specificity are hard to obtain.

Our assay was developed using the purified antibodies and proteins obtained. Our assay performance tests showed good results, with detection limits lower than 3 ng/mL, better than that of Roche's assay. Our predicted linearity range was set to 5–1200 ng/mL, the same as that used by Roche. In addition, our precision test was good, with no more than 8% CV. After the performance was verified, a method comparison was performed, which showed good correlation with Roche. The correlation coefficient R2 was 0.98 with a 95% confidence interval using the Passing–Bablok method.

Furthermore, despite the importance of these biomarkers, there are elusive conclusions of the roles of these markers in male patients. One study reported that the protocol for the Roche Elecsys total P1NP assay was used to determine the P1NP levels in 573 serum samples obtained from healthy female volunteers. The expected value for postmenopausal women is 16.27–73.87 ng/mL, compared to 15.13–58.59 ng/mL for premenopausal women. However, no male volunteers were included in the study. In our study, we reported that the expected value of P1NP for male was 23.36–77.05 ng/mL. This is the first time to establish the reference value of P1NP in men, which has a great guiding significance for the diagnosis and treatment of male osteoporosis.

Serum P1NP level could be significantly altered following the changes of the bone metabolism. The concentration of P1NP is normally much higher in infants and children than in adults. Serum P1NP is indictive for the disease activity in bone tissues in Paget's disease, in bone metastases of osteoblastic nature, and in the follow-up of osteoporosis treatment. For this reason, IFCC and IOF recommend using serum P1NP as a reference marker for bone formation in studies assessing fracture risk and treatment response. In previous studies, few have focused on the changes in P1NP or other bone loss markers in individuals after lung transplantation. In this study, we first collected serums from 19 patients with lung transplantation and examined P1NP level together with 25-VITD, OSTEOC, and *β*-CrossLap, as well as their BMDTs, every 3 months during follow-up of the transplantation. The results showed that P1NP, OSTEOC, and *β*-CrossLap were closely associated with BMDTs and bone loss. Analysis of the trend of bone markers in lung transplantation patients shows that *β*-CrossLap and P1NP are far more sensitive to bone formation and loss than BMDTs, especially when osteoporosis occurs rapidly. It was also found that 25-OH VD and OSTEOC were not a valid indicator of bone mass change in such a short period of time with very large changes in bone mass. Due to the sensitivity of P1NP and *β*-CrossLap to bone mass changes in patients after lung transplantation, P1NP and *β*-CrossLap are very good clinical markers for bone mass monitoring.

## Figures and Tables

**Table 1:** Summary table of frequency distribution.

Indicators	Results
Number of values	248
Mean ± SD	44.02 ± 15.58
Minimum; maximum	16.28; 104.6
Reference range^*∗*^	23.36–77.05
Lower 90% CI	21.02, 25.37
Upper 90% CI	69.35, 84.76

^*∗*^95% reference interval.

## Data Availability

The data used to support the findings of this study are available from the corresponding author upon request.

## Supplementary Materials

Supplementary Materials Supplementary Table 1: detection limit analysis with 3 lot of developed assays. Supplementary Table 2: precision analysis with 3 lot of developed assays. Supplementary Table 3: accuracy analysis with 3 lot of developed assays. .
