# Supplementary material for: Development of a Chemiluminescence Assay for Total N-Terminal Propeptide of Type I Collagen and Its Evaluation in Lung Transplantation
Source: J Anal Methods Chem. 2022 Jan 10;2022:2711414. doi: 10.1155/2022/2711414 (PMC8763551; doi:10.1155/2022/2711414)
Supplement: Supplementary Materials — Supplementary Table 1: detection limit analysis with 3 lot of developed assays. Supplementary Table 2: precision analysis with 3 lot of developed assays. Supplementary Table 3: accuracy analysis with 3 lot of developed assays. . [file 2711414.f1.docx]

**Supplementary Table 1: Detection limit analysis with 3 lot of developed assays.**

**
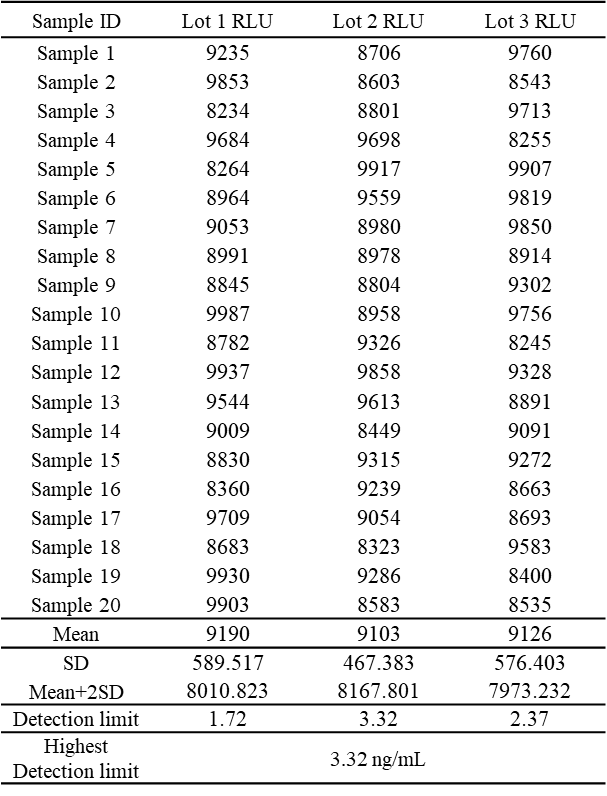
**

**Supplementary Table 2: Precision analysis with 3 lot of developed assays.**


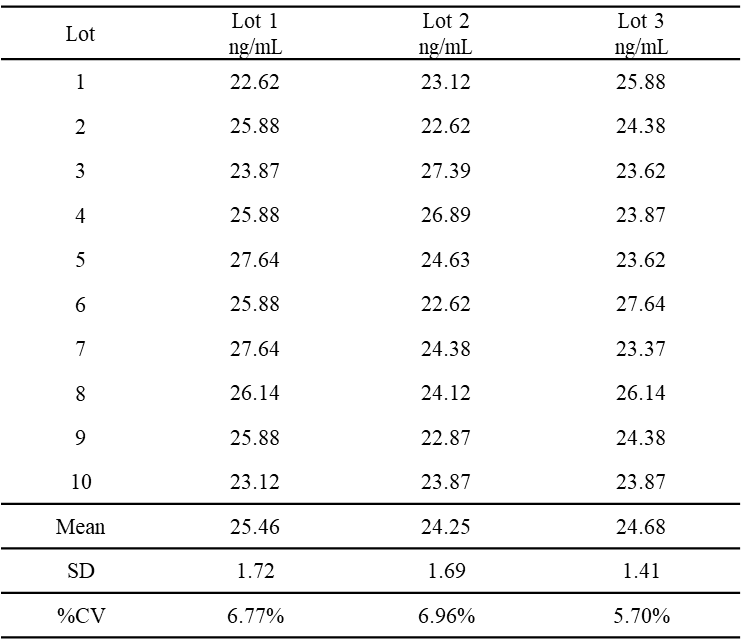


**Supplementary Table 3: Accuracy analysis with 3 lot of developed assays.**

| Lots | Theoretical value (ng/m) | Measured value（ng/mL） | | | |  |
| --- | --- | --- | --- | --- | --- | --- |
|  |  | Repeat 1 | Repeat 2 | Repeat 3 | Average | Bias |
| Lot 1 | 1124 | 1121.12 | 1183.2 | 1152.16 | 1152.16 | 2.69% |
|  | 474.67 | 469.92 | 455.68 | 498.41 | 474.67 | 4.58% |
|  | 158.22 | 156.32 | 161.26 | 176.07 | 164.55 | 6.25% |
|  | 52.74 | 51.17 | 49.11 | 54.79 | 51.69 | 5.56% |
|  | 13.19 | 12.28 | 13.03 | 12.28 | 12.53 | 3.46% |
| Lot 2 | 1124 | 1179.86 | 1139.27 | 1139.27 | 1152.8 | 2.03% |
|  | 474.67 | 503.38 | 513.35 | 478.47 | 498.4 | 3.60% |
|  | 158.22 | 176.07 | 169.3 | 162.53 | 169.3 | 4.00% |
|  | 52.74 | 57.43 | 55.11 | 61.49 | 58.01 | 5.57% |
|  | 13.19 | 15.23 | 14.79 | 13.48 | 14.5 | 6.28% |
| Lot 3 | 1124 | 1140.06 | 1152.05 | 1108.05 | 1133.39 | 2.01% |
|  | 474.67 | 498.59 | 488.71 | 493.65 | 493.65 | 1.00% |
|  | 158.22 | 156.55 | 159.62 | 144.27 | 153.48 | 5.29% |
|  | 52.74 | 56.85 | 60.33 | 56.85 | 58.01 | 3.46% |
|  | 13.19 | 13.06 | 13.06 | 13.45 | 13.19 | 1.71% |
